# Effect of Lactic Acid Fermentation on Volatile Compounds and Sensory Characteristics of Mango (*Mangifera indica*) Juices

**DOI:** 10.3390/foods11030383

**Published:** 2022-01-28

**Authors:** Juliana Mandha, Habtu Shumoy, Jolien Devaere, Joachim J. Schouteten, Xavier Gellynck, Ann De Winne, Athanasia O. Matemu, Katleen Raes

**Affiliations:** 1 Research Unit VEG-i-TEC, Department of Food Technology, Safety and Health, Campus Kortrijk, Ghent University, Sint-Martens-Latemlaan 2B, 8500 Kortrijk, Belgium; Juliana.mandha@UGent.be (J.M.); habtu.shumoy@UGent.be (H.S.); 2 Department of Food Biotechnology and Nutritional Sciences, Nelson Mandela African Institution of Science and Technology, P.O. Box 447, Arusha 23306, Tanzania; athanasia.matemu@nm-aist.ac.tz; 3 Centre for Aroma & Flavour Technology, Department of Microbial and Molecular Systems (M2S), Cluster Bioengineering Technology, KU Leuven, Technology Campus Ghent, Gebroeders De Smetstraat 1, 9000 Ghent, Belgium; jolien.devaere@kuleuven.be (J.D.); ann.dewinne@kuleuven.be (A.D.W.); 4 Department of Agricultural Economics, Ghent University, Coupure Links 653, 9000 Gent, Belgium; joachim.schouteten@UGent.be (J.J.S.); xavier.gellynck@UGent.be (X.G.)

**Keywords:** lactic acid bacteria, mango juice, fermentation, aroma, volatile profile, gas chromatography-mass spectrometry, sensory profile

## Abstract

Fermentation is a sustainable bio-preservation technique that can improve the organoleptic quality of fruit juices. Mango juices were fermented by monoculture strains of *Lactiplantibacillus plantarum* subsp. *plantarum* (MLP), *Lacticaseibacillus rhamnosus* (MLR), *Lacticaseibacillus casei* (MLC), *Levilactobacillus brevis* (MLB), and *Pediococcus pentosaceus* (MPP). Volatile compounds were sorbed using headspace solid phase microextraction, separated, and identified with gas chromatography-mass spectrometry. Forty-four (44) volatile compounds were identified. The control, MPP, and MLB had higher amounts of ethyl acetate, ethyl butyrate, 2-hexenal, 2,6-nonadienal, 2,2-dimethylpropanal, β-selinene, γ-gurjunene, α-copaene, and δ-cadinene, while MLC, MLP, and MLR had higher amounts of 2,3-butanedione and a cyclic hydrocarbon derivate. Consumers (*n* = 80) assessed their overall liking and characterized sensory attributes (appearance, color, aroma, flavor, consistency, acidity, and sweetness) using check-all-that-apply, and penalty analysis (just-about-right). Overall liking was associated with ‘mango color’, ‘pulp’, ‘mango aroma’, ‘sweet’, ‘natural taste’, and ‘mango flavor’ that described the control, MLB, MLC and MPP. Juices MLR and MLP were described as ‘bitter’, ‘sour’, ‘aftertaste’, and ‘off-flavor’. Multivariate analysis revealed relationships between the volatile compounds, mango juices fermented by different lactic acid bacteria, and sensory characteristics. Thus, the type of lactic acid bacteria strains determined the volatile and sensory profile of mango juices.

## 1. Introduction

Mango (*Mangifera indica* L.) is globally an important commercial fruit with high demand in the international market. It is among the top 10 major fruits cultivated in sub-Saharan Africa with a production of over 8 million tonnes/year [1]. Approximately 35% of produced fruits are lost post-harvest every year given it is a seasonal climacteric fruit with a few harvesting seasons, and its fresh fruit has a very short shelf-life. Thus, there is a need to transform this perishable fruit into products such as fruit juices with a long shelf-life and diversify its products through fermentation.

Fermentation using lactic acid bacteria increases food shelf-life by lowering pH and producing antagonistic metabolites such as organic acids and bacteriocins that are lethal to pathogens [2]. Furthermore, fermentation improves the nutritional and organoleptic quality of food [2]. Mango juice is often fermented to wine using yeasts (*Saccharomyces cerevisiae*) [3] but not as a non-alcoholic fermented juice. The few studies that investigated the use of lactic acid bacteria (*Lactobacilli*) as starter cultures in mango juice [4,5,6,7] lack an in-depth consumer study of the sensory acceptability of fermented non-alcoholic mango juices. Jin et al. [4] suggested consumer sensory evaluation as the next step to the formulation of non-alcoholic fermented mango juices.

Consumers choose food products by relying on sensory attributes and to ensure products’ success in the market, understanding and meeting their needs is vital. Rapid techniques such as check-all-that-apply (CATA) questions and penalty analysis using just-about-right (JAR) scales show important relationships between the samples and sensory characteristics [8]. Food processors, therefore, not only know how much consumers like a food product but also which sensory attributes drive consumer liking.

Food sensory characteristics are related to volatile and nonvolatile compounds which are often affected during processing [9]. During fermentation, microorganisms consume and/or produce volatile compounds in food that may change the overall sensory profile. These volatile changes are specific to microbial species because of their unique metabolic pathways related to their growth needs. Only a few studies have investigated the volatile compounds in lactic acid fermented mango juice. Moreover, this was reported in mango slurries with added sucrose [4] and glucose [10] after fermentation by *Lactiplantibacillus plantarum* subsp.* plantarum*, *Streptococcus thermophilus*, and *Lacticaseibacillus casei*. Further research without the addition of sugars and fermentation with other strains is required to bridge this research gap.

From literature [11,12,13,14,15], ten strains were selected for screening in mango juice. Mango juice was the sole raw material for microbial growth and metabolism, with no nutrient or pH adjustments. Hence, the selection criterion was based on growth or survival in mango juice in terms of viable cell counts (log CFU/mL) as monoculture strains after 24 h of fermentation. Results showed that among the ten strains—*Lactiplantibacillus plantarum* subsp. *plantarum* LMG6907, *Lacticaseibacillus rhamnosus* LMG25859, *Lacticaseibacillus casei* LMG6904, *Levilactobacillus brevis* LMG11437, *Pediococcus pentosaceus* LMG10740, *Lactobacillus acidophilus* LMG9433, *Lactobacillus johnsonii* LMG 24394, *Limosilactobacillus fermentum* LMG 8896, *Limosilactobacillus reuteri* LMG 9213, and *Leuconostoc mesenteroides* LMG6908—the highest growth was recorded in *Lactiplantibacillus plantarum* subsp. *plantarum, Lacticaseibacillus rhamnosus, Lacticaseibacillus casei, Levilactobacillus brevis*, and *Pediococcus pentosaceus* (Table S1).

Different strains of lactic acid bacteria metabolize nutrients depending on their specific transport (Spector & Alabama, 2009). *Lactiplantibacillus plantarum* subsp. *plantarum* and *Lacticaseibacillus rhamnosus* are facultative heterofermentative, *Lacticaseibacillus casei* and *Pediococcus pentosaceus* are predominately homofermentative while *Levilactobacillus brevis* is an obligate heterofermentative bacteria (Costa et al., 2019). Hence, they produce different types and quantities of metabolites and differently influence environmental characteristics such as pH and oxygen availability that may consequently affect the volatile and sensory profiles of fermented juices. In addition, different lactic acid bacteria strains differ in the specific activity of relevant enzymes involved in flavor formation during lactic acid fermentation (Smit, Smit, & Engels, 2005).

Therefore, we hypothesized that there is a discrepancy in the sensory and volatile profiles of mango juice fermented by different lactic acid bacteria, namely *Lactiplantibacillus plantarum* subsp. *plantarum, Lacticaseibacillus rhamnosus, Lacticaseibacillus casei, Levilactobacillus brevis*, and *Pediococcus pentosaceus*. The volatile compounds in the juices were measured by headspace solid phase microextraction (HS-SPME) combined with gas chromatography-mass spectrometry (GC-MS). Untrained consumers assessed the products’ sensory characteristics.

## 2. Materials and Methods

### 2.1. Mango Samples and Juice Preparation

Mango fruits (*Mangifera indica* L. var Kagoogwa) were purchased from Nakaseero market, in Kampala, Uganda (latitude: 00°18′42.34″ N, longitude: 32°34′46.34″ E). The fruits were selected based on their maturity, uniform color, no visible infection, and no mechanical damage. They were washed using distilled water, peeled, chopped, and mixed using a domestic blender (Joseph, MI, USA) to obtain mango juice without the addition of water. The obtained mango juice was homogenized using an Ultra-Turrax (IKA T18, Staufen, Germany) at 1422× *g* (10,000 rpm) for 15 min and pasteurized according to Shaheer et al. [16]. Briefly, 50 mL of mango juice dispensed in a sterile 100 mL flask was pasteurized at 80 °C (internal temperature) for 5 min in a water bath (Memmert WNB 45, Schwabach, Germany) with an external temperature of 100 °C under continuous shaking. The pasteurized juice was rapidly cooled to room temperature using an ice-water bath (0 °C).

### 2.2. Lactic Acid Bacteria Strains and Growth Conditions

Lactic acid bacteria strains (*Lactiplantibacillus plantarum* subsp. *plantarum*, LMG6907, *Lacticaseibacillus rhamnosus* LMG25859, *Lacticaseibacillus casei* LMG6904, *Levilactobacillus brevis* LMG11437, and *Pediococcus pentosaceus* LMG10740) were purchased from the Belgian Coordinated Collections of Microorganisms-Laboratory of Microbiology (BCCM-LMG, Ghent, Belgium). The dried cultures were grown in sterile de Man, Rogosa, and Sharper (MRS) broth (Basingstoke, Hampshire, England) and stored in cryovials with 20% *v*/*v* glycerol (VWR International, Leuven, Belgium) at −20 °C.

Before use, each strain was activated twice in MRS broth at 30 °C (*Lactiplantibacillus plantarum* subsp. *plantarum, Levilactobacillus brevis*, and *Pediococcus pentosaceus*) and 37 °C (*Lacticaseibacillus casei* and *Lacticaseibacillus rhamnosus*) for 24 h (stationary growth phase). The cultures were centrifuged (Hermle Z300K, Hermle Labortechnik GmbH, Wehingen, Germany) at 2540× *g* (4000 rpm) for 15 min at 4 °C, the biomass washed twice in saline diluent (0.85% *w*/*v* sodium chloride, VWR International, Belgium) and then re-suspended in the same diluent [17].

### 2.3. Fermentation of Mango Juice

Pasteurized mango juices were inoculated with monoculture washed bacterial cells (1% *v*/*v*) and incubated at optimal growth temperatures (30 or 37 °C) for 24 h to obtain mango juice fermented by *Levilactobacillus brevis* (MLB), *Lacticaseibacillus casei* (MLC), *Lacticaseibacillus rhamnosus* (MLR), *Lactiplantibacillus plantarum* subsp. *plantarum* (MLP), and *Pediococcus*. *pentosaceus* (MPP). The control was pasteurized mango juice without the addition of lactic acid bacteria and incubated under the same conditions (30 °C, 24 h). The juices were then kept at 4 °C to stop fermentation and analyzed within 12 h. Three independent fermentation experiments were carried out for each bacterial strain.

### 2.4. Growth of Microorganisms and pH during Fermentation

Viable bacterial counts were enumerated from the samples at time zero (T_0_) and after 24 h (T_24_). Each sample (1 mL) was vortex (Vortex-genie 2, Thermo Fisherc Sientific Inc., Waltham, MA, USA) mixed in 9 mL of sterile saline diluent and serial dilutions (10^−1^–10^−7^) were subsequently plated (0.1 mL) using the spread plate method on MRS agar, Rose Bengal chloramphenicol agar, plate count agar, Xylose Lysine Deoxycholate agar, and Rapid *E. coli* agar (Oxoid LTD, Basingstoke, Hampshire, England). The plates were incubated at optimal growth conditions for the enumeration of *Lactobacillus* (30/37 °C, 48 h), yeast and molds (20 °C, 5 days), total plate counts (20 °C, 3 days), *Salmonella* (37 °C, 24 h), *Escherichia coli* (44 °C, 24 h), and total coliforms (37 °C, 24 h) [18].

pH was measured using a digital pH meter (FC 2020) at 20 °C, previously calibrated with buffer solutions (4, 7, and 10).

### 2.5. Analysis of Volatile Compounds

#### 2.5.1. Extraction of Volatiles

Volatile compounds in the samples were analysed using HS-SPME GC-MS according to a method described by Hinneh et al. [19] with some modifications. Briefly, 2 g of juice sample was added to each HS-SPME vial (20 mL) and thoroughly mixed with 2 mL of saturated sodium chloride (NaCl) (Merck, Belgium) previously brought to pH 3.0 (with 0.8 M acetic acid solution, Merck, Belgium) and 3 µL of the internal standard, 2-octanol (Sigma-Aldrich, Belgium) at a concentration of 213.6 mg/L methanol (Merck, Belgium). The vial was hermetically sealed and then incubated (Gerstel, Müllheim an der Rur, Germany) at 40 °C for 20 min in a thermostatic agitator to extract the volatiles. The released volatiles in the headspace were subsequently sorbed onto the divinylbenzene/carboxen/polydimethylsiloxane (DVB/CAR/PDMS) fiber (75 µm, Sigma-Aldrich, Belgium) for 20 min at 40 °C. Three independent experiments for each lactic acid fermentation were carried out and between each GC-MS analysis, the fiber was conditioned for 7 min at 270 °C.

#### 2.5.2. GC-MS Analysis

The gas chromatograph (GC) (Agilent 6890, Agilent Technologies, Santa Clara, CA, USA) was connected to a mass spectrometer fitted with a ZB-Wax plus column (30 m × 0.25 mm i.d. × 0.25 µm film thickness, Zebron, Phenomenex, Macclesfield, UK). Helium gas was used as a carrier gas with a constant flow rate of 1 mL/min. The DVB/CAR/PDMS fiber was inserted and desorbed for 180 s into the splitless injection port (250 °C) of the GC oven. The following time-temperature program was applied: 40 °C for 5 min, then increased at 5 °C/min to 80 °C, at 3 °C/min to 134 °C, and at 8 °C/min to 230 °C, where it was held for 2 min. Mass spectrometry was performed at a 230 °C ion source temperature with a mass range from *m/z* 40 to 300 (full scan mode) and 70 eV ion current using no solvent delay and a threshold of 50 [20]. The extracted volatile compounds detected were identified.

#### 2.5.3. Identification of Volatile Compounds

Volatile compounds were identified by comparing retention indices on the ZB-Wax-column with literature data, matching the MS-spectrum of each peak to those of the Wiley275 library (quality match > 85%), and their retention index (RI) values were calculated using a series of n-alkanes (C_9_–C_16_) as standards according to Vandendool & Kratz [21]. An internal standard method was used to quantify the identified volatiles [22]. Therefore, data have been expressed as nanograms of the internal standard (2-octanol) equivalents per mL of sample and were calculated as:

(1) $$M_{s}{=(M}_{i}{\;\times \;A}_{s})\;/{(A}_{i}{\;\times \;M}_{o}).\;$$

where $M_{s}$ is the identified volatile concentration, expressed as ng/mL; $M_{i}\;$ is the weight of the internal standard, expressed as ng; $M_{o}$ is the weight of mango juice used, expressed as mL; $A_{s}$ is the peak area of identified volatiles; and $A_{i}$ is the peak area of the internal standard. Percentage differences of the volatiles in each fermented mango sample versus the control were calculated to evaluate any differences between the samples.

### 2.6. Consumer Sensory Acceptability

#### 2.6.1. Participants

Eighty (80) participants were randomly recruited from students, staff, and visitors of the Nelson Mandela African Institution of Science and Technology. Eligibility for participation followed the criteria of Meilgaard et al. [23]: no food allergies (oral allergy syndrome) or dietary intolerances, consumption of fruits, willingness, and availability. All participants’ demographics are described in Table S2. The majority were aged between 18 and 49 years with 55% males and 45% females. No prior information regarding the aim of the study or content of the products was given, and no reimbursements were made for their participation.

This study was approved by the Health Research Ethics Committee of Kibong’oto Infectious Diseases Hospital, the Nelson Mandela African Institution of Science and Technology, and the Centre for Educational Development in Health Arusha under the protocol number KNCHREC0008, and each participant gave informed consent for inclusion before they participated in the study.

#### 2.6.2. Sensory Data Collection

Juice samples (20 mL) were served cold (4 ± 1 °C) in styrofoam cups identifiable by a random three-digit code. Each consumer received six samples (5 fermented and control juices) one at a time and between each different sample, two unsalted crackers and bottled water were provided to rinse their mouths, and a 2 min break was taken. The samples were served in a completely randomized order using William’s Latin square design [24] to balance bias caused by first-order and carry-over effects. This experiment took place in a room with a classroom arrangement, adequate lighting, noise-free uninterrupted environment, and participants did not face each other.

For each sample, consumers first rated their overall liking using a 9-point hedonic scale [25] with 1 = ‘dislike it extremely’, and 9 = ‘like it extremely’. This hedonic scale also assessed the appearance, aroma, sweetness, flavor, consistency, acidity, and color attributes of the juices.

Secondly, consumers used the check-all-that-apply (CATA) method [26] to characterize the samples. This is a multi-choice question that comprises a list of terms from which the consumers select. The terms used were based on prior work [27] and included: ‘mango aroma’, ‘mango color’, ‘mango flavor’, ‘thick’, ‘pulp’, ‘sweet’, ‘sour’, ‘off-flavor’, ‘natural taste’, ‘intense flavor’, ‘light color’, ‘bitter’, and ‘aftertaste’. Consumers were requested to check all applicable terms. For each sample, these terms were randomized in a monadic sequence following a balanced order by using William’s Latin square design [24].

Thirdly, consumers assessed modifiable attributes of the sample, i.e., aroma, sweetness, flavor, consistency, acidity, and color using a 5-point just-about-right (JAR) scale, anchored from 1 = ‘much too low’ to 5 = ‘much too high’ [28].

Finally, consumers stated their intent on whether they would likely purchase the product in the market using a 5-point scale ranging from ‘certainly would not buy’ to ‘certainly would buy’ [29]. Consumers were also asked questions regarding their age, gender, frequency of fruit consumption per month (‘more than once a week’, ‘once a week’, ‘more than once a month but less than every week’ or ‘less than once a month’) [30], and whether they paid attention to their diet.

### 2.7. Statistical Analysis

Statistical analyses were performed using XL-STAT, (version 2020.1, Addinsoft, Paris, France), IBM SPSS for macOS (Version 23, IBM Corporation, Armonk, New York, NY, USA), and GraphPad Prism (Version 8.0.0 for macOS, San Diego, CA, USA). All the microbiology and volatile assays were performed in triplicates in three independent experiments, and results were expressed as the assay’s average. Data of volatile compounds were analyzed using one-way analysis of variance (ANOVA) followed by a post-hoc Tukey *t*-test to determine any significant differences (*p* < 0.05) between samples. Principal component analysis (PCA) was used to study relationships between samples in terms of volatile profiles.

For the sensory data, repeated measures one-factor ANOVA and Bonferroni post-hoc test was used to check for differences in the overall liking and sensory attributes between the different samples. A frequency analysis assessed attributes on the JAR scale and thereafter penalty analysis [31] examined if any of the attributes influenced a mean drop in the overall acceptability for each sample. Based on Pareto’s principle, significant (*p* < 0.05) results were considered when a proportion of >20% consumers criticized an attribute either as too ‘low’ (−) or too ‘high’ (+) and caused a mean drop of >1 point on overall liking [28]. CATA data were analyzed using Cochran’s Q test [32], which analyses a two-way randomized block design (data matrix) to check if the samples as treatments have similar effects (McNemar post-hoc) when the consumer response is binary (checked/not checked) [8].

A multiple factorial analysis (MFA) [33] was used to determine relationships between the samples based on the overall liking, liking of key sensory attributes, CATA characteristics, and volatiles data.

## 3. Results

### 3.1. Growth of Lactic Acid Bacteria in Mango Juice during Fermentation

The mango juices had an initial (T_0_) lactic acid bacteria concentration of 7–8 log CFU/mL, but after 24 h fermentation (T_24_), the viable counts increased to a maximum of 9.16 log CFU/mL in MLB (Table 1). This increment was significant (*p* < 0.05) in MLB, MLP, and MLR. Counts in the control were below the detectable limit of <1 log CFU/mL.

The microbial analysis also showed that the control had a total plate count (<3 log CFU/mL) and yeast and molds (<2 log CFU/mL) below permitted levels (<4 and <3 log CFU/mL, respectively) according to the Codex Alimentarius Commission of the Food and Agricultural Organization [34]. Similarly, besides the lactic acid bacteria, no other microorganisms were observed using total plate count (<3 log CFU/mL) and yeast and mold were below detectable limits (<2 log CFU/mL) in the samples. Total coliforms in the samples were below detectable limits (<1 log CFU/mL), as well as pathogenic microorganisms *Escherichia coli* and *Salmonella* spp. (<1 log CFU/mL).

The initial pH of the mango juice was 4.45 ± 0.13. The control juice remained at the same pH (4.39 ± 0.12) after 24 h incubation. However, for the fermented juices, pH significantly decreased in MLC (4.09 ± 0.14, *p* = 0.03), MPP (3.94 ± 0.14, *p* = 0.009), MLB (3.83 ± 0.10, *p* = 0.003), MLR (3.81 ± 0.20, *p* = 0.01), and MLP (3.72 ± 0.19, *p* = 0.005).

### 3.2. Analysis of Volatile Compounds

Forty-four (44) volatile compounds were tentatively identified in the samples (Table 2) and classified into different groups: monoterpenes (16), sesquiterpenes (13), esters (4), alcohols (3), aldehydes (3), hydrocarbons (1), furans (1), sulfurs (1), trihalomethane (1), and ketones (1).

**Figure 1:**
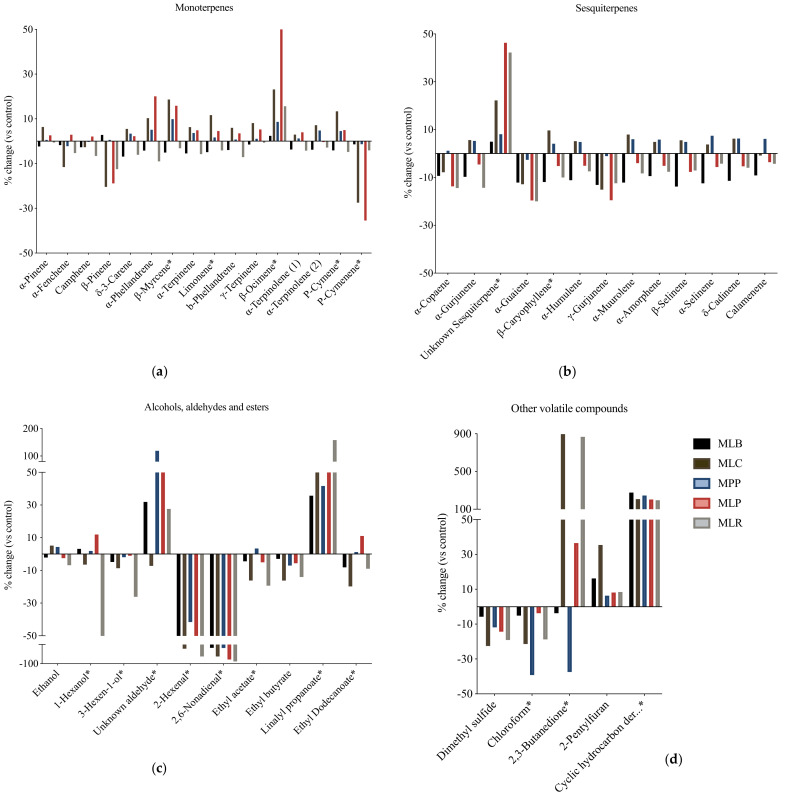
Percentage change of volatile compounds (ng/mL of 2-octanol equivalents). Mango juice fermented with MLB—*Levilactobacillus brevis*; MLC—*Lacticaseibacillus casei*; MLP—*Lactiplantibacillus plantarum* subsp. *plantarum*; MLR—*Lacticaseibacillus rhamnosus*; MPP—*Pediococcus pentosaceus*. Control is mango juice with no lactic acid bacteria under the same conditions of fermentation (24 h). * Significant difference (*p* < 0.05). (**a**) Monoterpenes; (**b**) Sesquiterpenes; (**c**) Alcohols, aldehydes, and esters; (**d**) Other volatile compounds.

In the control sample, mainly monoterpenes were detected: δ-3-carene, α-pinene, β-myrcene, α-terpinene, limonene, and β-phellandrene (Figure S1). After fermentation (24 h), some variations in volatile concentrations were observed, for instance, the levels of 2,6-nonadienal (cucumber notes) and 2-hexenal (apple and green notes) fell sharply in all samples while a cyclic hydrocarbon derivate, originally not in the control, was detected in all fermented juices (Table 2). The percentage change (%) of volatile compounds in fermented mango juices compared to the control was therefore calculated (Figure 1). Representation of volatile concentrations −100% mean complete degradation and +100% mean production after fermentation.

The total level of the monoterpenes (Figure 1a) did not significantly change (<15%) after fermentation. However, there was a significant increase in β-ocimene in MLP (*p* = 0.001), limonene in MLC (*p* = 0.010), and β-myrcene in MLP and MLC (*p* = 0.004) while the p-cymene decreased (*p* = 0.033) in MLC and MLP. Most of the sesquiterpenes decreased (Figure 1b) in MLB, MLP, and MLR except for an unknown sesquiterpene which increased in MLP and MLR by over 40% (*p* < 0.05). In MPP and MLC, a slight increase (<10%) in the total level of sesquiterpenes was observed. Especially for the level of β-caryophyllene, a significant (*p* < 0.05) decrease was recorded in MLB and MLR. In “other volatiles” (Figure 1d), a cyclic hydrocarbon derivate was unique to fermented juices with a production of >+100% after fermentation and was not detectable in the control. The chemical structures of the terpene compounds are shown in Table S3.

Alcohol concentrations (Figure 1c) significantly decreased in MLR by 63% in 1-hexanol (*p* ≤ 0.001) and 26.1% in 3-hexen-1-ol (*p* = 0.027). Unsaturated aldehydes 2,6-nonadienal and 2-hexenal were degraded by more than 80% and 42% after fermentation in all the fermented juices, but the concentration of an unknown aldehyde significantly increased (*p* = 0.006) in MPP.

Four (4) esters were found in the samples. After fermentation, in all the fermented juices, the levels of ethyl butyrate and ethyl acetate (except MPP) decreased while concentrations of linalyl propanoate significantly increased (35–158%). Among other volatiles (Figure 1d), the amount of 2,3-butanedione increased (*p* < 0.05) tremendously in MLC and MLR juices by 282% and 419%, respectively, as opposed to MPP and MLB where it significantly decreased (*p* < 0.05) after fermentation.

**Figure 2:**
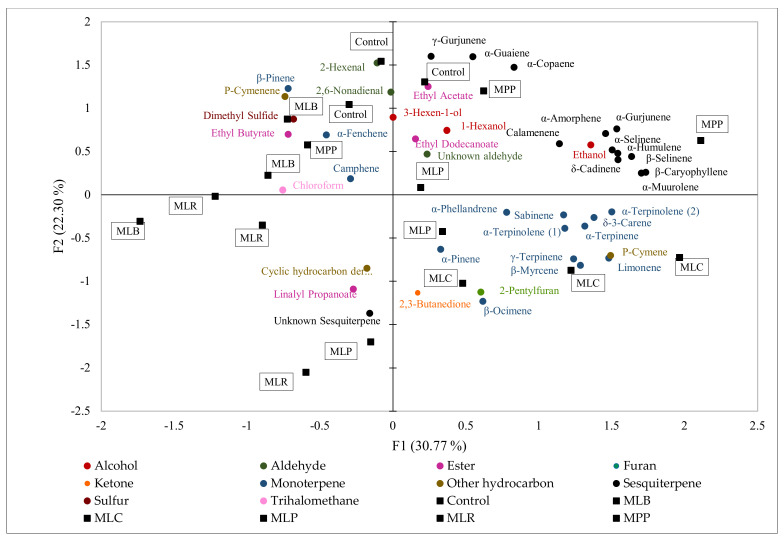
Principal component analysis (PCA). Biplot based on values of volatile compounds (ng/mL of 2-octanal equivalents) in mango juice. Mango juices fermented with MLB—*Levilactobacillus brevis*; MLC—*Lacticaseibacillus casei*; MLP—*Lactiplantibacillus plantarum* subsp. *plantarum*; MLR—*Lacticaseibacillus rhamnosus*; MPP—*Pediococcus pentosaceus*. Control is mango juice with no lactic acid bacteria under the same conditions of fermentation (24 h).

The relationships among the samples based on their volatile data were illustrated using a principal component analysis (PCA) plot (Figure 2). Considering the 44 compounds, the first two PCA dimensions accounted for 30.8% (PC1) and 22.3% (PC2) of the variance. PC1 separated MLC from MLR, but the results showed PC2 was the main axis for the separation of the control from MLC, MLR, and MLP. The control, MPP, and MLB were localized on the PC2 positive semi-axis due to higher levels of esters (ethyl acetate, ethyl butyrate), aldehydes (2-hexenal, 2,6-nonadienal, and an unknown aldehyde), and sesquiterpenes (β-selinene, γ-gurjunene, α-copaene, and δ-cadinene). MLC, MLP, and MLR were localized on the PC2 negative semi-axis and had higher amounts of 2,3-butanedione, an unknown sesquiterpene, and a cyclic hydrocarbon derivate.

### 3.3. Consumer Sensory Acceptability

All the samples were liked moderately ranging from 7.71 in the control to 6.71 in MLR (Table 3). The fermented mango juices did not differ significantly (*p* > 0.05) from the control except for MLR, which was rated significantly lower. Although aroma and flavor were most liked in MLB (7.63 and 7.66, respectively), MLB was similar to the control, MLC, and MPP in these attributes. However, MLP and MLR juices received the lowest scores (6.28–6.96) and differed (*p* < 0.05) from the control in terms of aroma, flavor, consistency, and sweetness.

**Figure 3:**
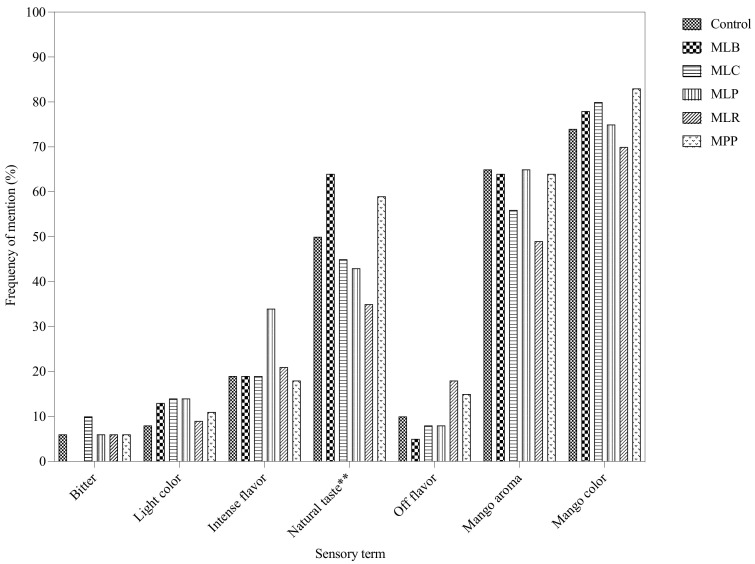

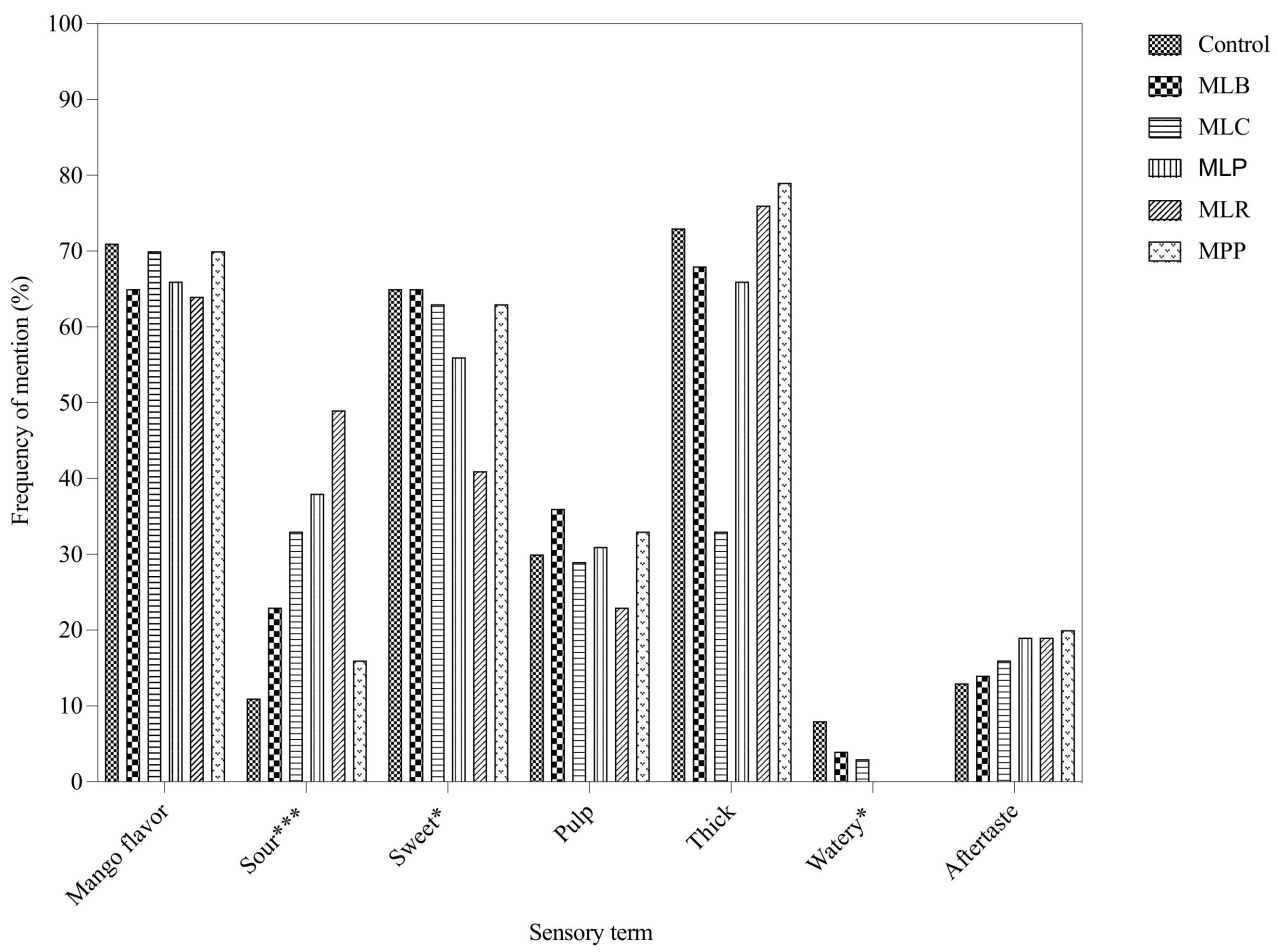
Frequency of mention (% respondents, *n* = 80) of CATA terms. Mango juices fermented with MLB—*Levilactobacillus brevis*; MLC—*Lacticaseibacillus casei*; MLP—*Lactiplantibacillus plantarum* subsp. *plantarum*; MLR—*Lacticaseibacillus rhamnosus*; MPP—*Pediococcus pentosaceus*. Control is mango juice with no lactic acid bacteria under the same conditions of fermentation (24 h). Significant differences * *p* < 0.05, ** *p* < 0.01, *** *p* < 0.001 using Cochran’s Q test.

The CATA question obtained binary responses of terms that consumers perceived to describe the samples. The terms ‘mango flavor’, ‘mango color’, ‘mango aroma’, ‘sweet’, ‘thick’, and ‘natural taste’ were most frequently (>60% of consumers) used to describe the samples (Figure 3). Fermented juices did not differ from the control in most of the sensory terms but 4 out of 14 terms were significantly different, i.e., ‘natural taste’ (*p* = 0.003), ‘sour’ (*p* = 0.001), ‘sweet’ (*p* = 0.011), and ‘watery’ (*p* = 0.008). MLB had the highest mention of ‘natural taste’ at 64% followed by MPP (59%) and the control (50%), whereas MLR had the highest mention of ‘sour’ (49%) and least mention of ‘sweet’ (41%).

**Figure 4:**
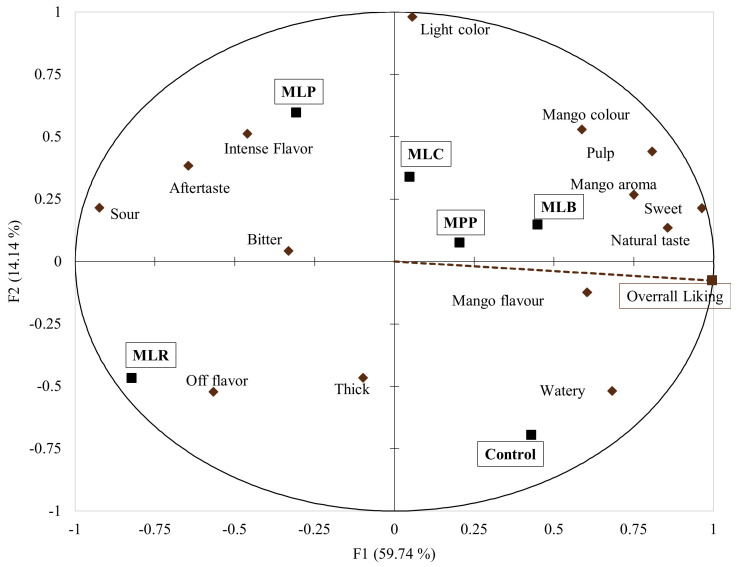
Multifactorial analysis (MFA) using overall liking ratings and CATA (*n* = 80). Mango juices fermented with MLB—*Levilactobacillus brevis*; MLC—*Lacticaseibacillus casei*; MLP—*Lactiplantibacillus plantarum* subsp. *plantarum*; MLR—*Lacticaseibacillus rhamnosus*; MPP—*Pediococcus pentosaceus*. Control is mango juice with no lactic acid bacteria under the same conditions of fermentation (24 h).

A sensory map from multi-factorial analysis (MFA) evaluated whether the allocation of these terms contributed to overall liking and showed any relationships between the product categories. The first two MFA dimensions (Figure 4) explained 73.9% of the total variability. There was a good correlation between different samples, sensory terms, and overall liking. The positive F1 semi-axis represented the control, MLB, MPP, and MLC. These juices were closely associated with overall liking and characterized with ‘mango color’, ‘pulp’, ‘mango aroma’, ‘sweet’, ‘natural taste’, and ‘mango flavor’ terms. Conversely, MLP and MLR juices were in the negative F1 semi-axis (separate level) and characterized with ‘sour’, ‘bitter’, ‘aftertaste’, and ‘off-flavor’ terms.

**Figure 5:**
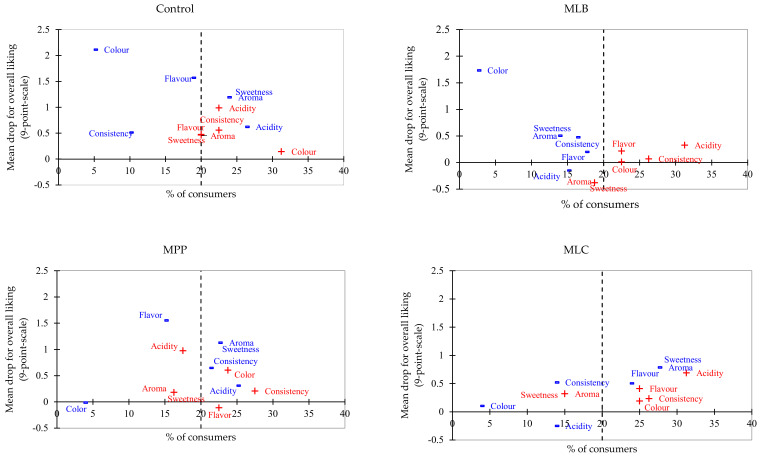

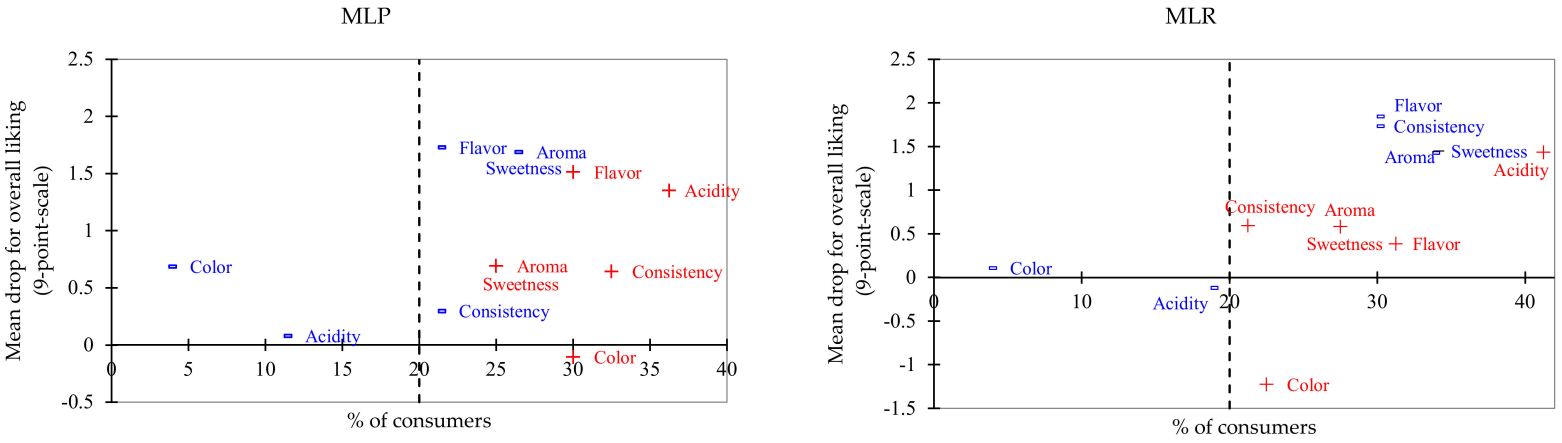
Penalty analysis. A proportion of >20% of consumers (indicated by the --- line) who considered an attribute too ‘low’ (−) or too ‘high’ (+) and caused a mean drop of >a point was considered significant (*p* < 0.05). (*n* = 80). Mango juices fermented with MLB—*Levilactobacillus brevis*; MLC—*Lacticaseibacillus casei*; MLP—*Lactiplantibacillus plantarum* subsp. *plantarum*; MLR—*Lacticaseibacillus rhamnosus*; MPP—*Pediococcus pentosaceus*. Control is mango juice with no lactic acid bacteria under the same conditions of fermentation (24 h).

Penalty analysis obtained information on the intensity level of modifiable sensory attributes of each sample. Overall, consumers who found the samples to deviate from just-about-right was less than 50% (Figure 5). Moreover, attributes that fell in the upper right corner were considered most concerning as they have the highest skews and had the greatest mean drop, while those in the lower-left corner are those with minimal concern. As observed, the control, MLB, MPP, and MLC had the least penalized attributes in the upper right corner compared to MLR and MLP. No sensory attribute exceeded the 20% threshold for MLB. The control and MPP had only two out of six attributes above the threshold, MLC registered three attributes, MLP had four attributes, while for MLR, all six attributes were criticized by > 20% of consumers.

Sweetness and aroma were the most penalized attributes and were considered too low in the control, MPP, MLC, MLR, and MLP, causing a mean drop for overall liking scores ranging from 1.10 in MLC to 1.69 in MLP juice. Acidity was penalized for being too high in MLC, MLR (mean drop 1.43), and MLP (mean drop, 1.35). Consumers had conflicting opinions on the flavor of MLP as 21.3% found it to be too high (1.73 mean drop) and 30% too low (1.51 mean drop). Consistency and color were only criticized in MLR as too low and too high, respectively.

Regarding consumers’ purchase willingness, the control had the highest percentage of ‘certainly would buy’ at 46% followed by MLB and MPP at 40% (Figure S2). The highest scores for MLR and MLP juices were recorded at ‘might buy’ at 32.5% and 33.75%, respectively.

### 3.4. Combination of Volatile and Sensory Analysis

**Figure 6:**
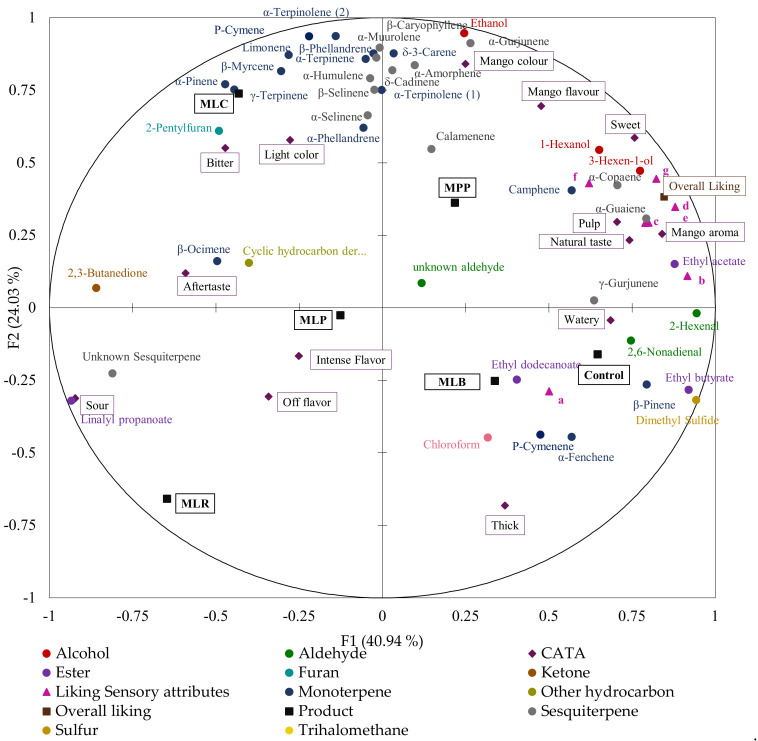
Multifactorial analysis (MFA) showing the relationship between the volatile compounds (ng/mL of 2-octanol equivalents), overall liking, liking of sensory attributes ratings, and CATA characterization. Mango juices fermented with MLB—*Levilactobacillus brevis*; MLC—*Lacticaseibacillus casei*; MLP—*Lactiplantibacillus plantarum* subsp. *plantarum*; MLR—*Lacticaseibacillus rhamnosus*; MPP—*Pediococcus pentosaceus*. Control is mango juice with no lactic acid bacteria under the same conditions of fermentation (24 h). Liking of sensory attributes, a = liking color, b = liking consistency, c = liking sweetness, d = liking flavor, e = liking aroma, f = liking appearance, and g = liking acidity. (*n* = 80).

Volatile data were compared with sensory data (CATA, sensory liking of the attributes and overall liking) using multifactorial analysis (Figure 6). The first two dimensions of MFA explained 65% of the total variability. The positive axis of the first dimension (F1) separated the control, MLB, and MPP and associated them with overall liking, desirable CATA terms (‘sweet’, ‘mango flavor’, ‘mango color’, ‘natural taste’, and ‘pulp’), liking of sensory attributes, alcohols, aldehydes, esters (ethyl butyrate), some sesquiterpenes (γ-gurjunene, α-copaene), and some monoterpenes (camphene, α-fenchene). Contrarily, MLR, MLP, and MLC juices were on the negative axis of F1 which was associated with the CATA terms ‘sour’, ‘off-flavor’, ‘intense flavor’, ‘aftertaste’, and ‘bitter’, 2,3 butanedione, linalyl propanoate, 2-pentylfuran, some monoterpenes (limonene, β-myrcene, β-ocimene), and some sesquiterpenes (α-humulene, α and β-selinene).

## 4. Discussion

Lactic acid bacteria grew and survived in mango juice, and this may be attributed to nutrients (carbohydrates, organic acids, vitamins, and minerals) in mango that are a source of energy for metabolism. Other studies have also demonstrated mango as a suitable medium for lactic acid bacteria growth [4,5,6,7]. After fermentation, the acidity of fermented mango juices increased, and this may be antagonistic to pathogenic microorganisms increasing juice shelf-life.

Monoterpenes and sesquiterpenes were the key compounds in the mango juices unlike our previous study in watermelon juices [35]. α-Terpinolene, 3-carene, and limonene have also been identified as key monoterpenes in Australian mango cultivars [36] and mango pulp [37]. The presence of monoterpenes in both the control and fermented samples could have played a significant role in consumer acceptability, i.e., fermentation of mango juice with *Levilactobacillus brevis, Lacticaseibacillus casei, Lactiplantibacillus plantarum* subsp. *plantarum*, and *Pediococcus. pentosaceus* except *Lacticaseibacillus rhamnosus* did not affect overall liking (acceptability). Monoterpenes give characteristic flavor notes; turpentine, sweet, and fruity notes. However, the significant increase in β-ocimene (MLP and MLC), limonene (MLC), and β-myrcene (MLP and MLC) (Table 2) may contribute a slightly irritating odor [10]. Sesquiterpenes may contribute to juice flavors and the detected sesquiterpenes were β-selinene, α-gurjunene (woody), α-copaene (woody, spice), and δ-cadinene (woody, thyme). Oliver-Simancas et al. [37] reported β-caryophyllene and α-humulene as the most abundant sesquiterpenes in fresh mango pulp. The flavor and aroma of the control, MLB, MLC, and MPP were most liked and least liked in MLP and MLR (Table 3).

During fermentation, lactic acid bacteria produce various products that may directly or indirectly be involved in the decrease or increase in volatile compounds. Although the total level of the monoterpenes did not significantly change after fermentation, a significant increase was observed in β-ocimene, limonene, and β-myrcene. Lactic acid bacteria produce acids such as lactic acid and acetic acid during fermentation that may damage the fruit cells leading to the release of these compounds [38]. In addition, it is generally recognized that monoterpenes originate from the plastids of pyruvate and glyceraldehyde-3-phosphate via the 2-*C-*methyl-d-erythritol 4-phosphate (MEP) pathway [39]. Lactic acid bacteria possess an extensive array of enzymes including terpene synthases, which can be produced by *Levilactobacillus brevis* and *Pediococcus pentosaceus* and are involved in their biosynthesis and biochemical reactions [40]. On the other hand, most sesquiterpenes decreased (Figure 1b) after fermentation, which is in agreement with Park et al. [41] who also found that lactic acid bacteria significantly decreased terpenes in a mixed berry juice. This reduction could be due to their oxidation to secondary products, hydroxylation, acylation, or isomerization [39,42].

2,3-Butanedione was the only ketone detected. Lactic acid bacteria have plasmid-encoded citrate transporter genes and together with enzyme citrate lyase, they can degrade citrate present in mango to 2,3-butanedione [43]. Strains such as *Lacticaseibacillus casei, Lacticaseibacillus rhamnosus*, and *Lactiplantibacillus plantarum* subsp. *plantarum* can convert citric acid during citric acid metabolism to acetate and oxaloacetate under the catalysis of citric acid lyase. The oxaloacetate is decarboxylated by oxaloacetate decarboxylase to produce pyruvate [44]. The pyruvate is then condensed by α-acetolactate synthase to α-acetolactate, which is chemically unstable and can be converted to diacetyl (2,3-butanedione) in a non-enzymatic oxidative decarboxylation reaction or by α-acetolactate decarboxylase [45]. After fermentation, 2,3-butanedione tremendously increased by >800% in MLC and MLR (Figure 1d). *Lacticaseibacillus rhamnosus* produces high amounts of 2,3-butanedione (64 mg/g glucose) [46] which have a profound effect on the flavor and aroma of fermented products as it is characterized by a strong buttery odor that may probably not be organoleptically acceptable. Hence, 2,3-butanedione may be an index for product quality control. Another compound, i.e., 2-pentylfuran, which has odor notes of beany, oxidized, and green could also give an undesirable buttery flavor to the sample of MLC (Figure 2).

Strong fruity aromas such as apple-like (ethyl butyrate) and pineapple-like notes (ethyl acetate) were also found in the samples. After fermentation, their levels decreased (Table 2) unlike in other fruit juices such as apple juice, where a slight increase after *Lactiplantibacillus plantarum* subsp.* plantarum, Lacticaseibacillus rhamnosus*, and *Lacticaseibacillus casei* fermentation was reported [47]. Linalyl propanoate, which gives citrus-like notes, significantly increased (35–158%) in all fermented juices (Table 2). This aliphatic (straight-chain) ester may be formed from the metabolism of fatty acids through β-oxidation.

Aldehydes (2-hexenal and 2,6-nonadienal) could also influence sample sensory attributes as they give fatty-grassy and cucumber notes, respectively. Liu et al. [48] also identified 2,6-nonadienal in fresh Tianong mango pulp. After fermentation, 2-hexenal and 2,6-nonadienal were degraded. This result is in agreement with Jin et al. [4] who reported a decrease in aldehydes in mango slurries fermented by *Lactiplantibacillus plantarum* subsp. *plantarum*. During fermentation, aldehydes may be reduced to their corresponding alcohols or oxidized to acids [49]. A high level of aldehydes may cause off-flavors whichnegatively impact the sensory characteristics of fermented food [50].

Sweetness and consistency were most liked in the control, MLB, MLC, and MPP, while MLP and MLR were the least scored. This correlated with volatile analysis which showed that δ-3-carene, with sweet and limonene-reminiscent odor responsible for ripe mango flavor [51], were highly concentrated in MLC while β-ocimene, responsible for the warm, herbaceous, and floral odor characteristic of raw (unripe) mango flavor was mainly present in MLP, MLR, and MLC (Figure 6). MLR had a significant decrease in the concentration of alcohols (Table 2); 1-hexanol (fruity and aromatic flavor) and 3-hexen-1-ol (intense green grassy odor) that give desirable sweet flavor notes.

The CATA data showed that ‘mango flavor’, ‘mango color’, ‘mango aroma’, ‘sweet’, ‘thick’, and ‘natural taste’ (Figure 3) were the main drivers of consumer liking as they were the most frequently used terms. Thus, consumers like fermented mango juices that still maintain the natural taste and flavors of mango juice. Consumers also detected differences between the samples, as there were significant differences in the frequency of mention using Cochran’s Q test (*p* < 0.05) similar to other studies that characterized orange juices [52] and chocolate milk deserts [26]. The MFA (Figure 4) showed relationships between the samples based on their CATA characteristics, overall liking, liking of key sensory attributes, and volatiles. Overall liking was strongly associated with the control, MLB, and MPP which were characterized by ‘mango aroma’, ‘natural taste’, ‘sweet’, and ‘mango flavor’ terms, sesquiterpenes, monoterpenes, alcohols, and aldehydes. MLP, MLR, and MLC were associated with ‘off flavor’, ‘sour’, ‘aftertaste’, and ‘intense flavor’ terms probably due to high levels of 2,3-butanedione.

Regarding penalty analysis, different attributes had a differential effect on the overall acceptability of each product. MLB was highly accepted as none of its attributes led to a mean drop in overall acceptability (Figure 5). Its attributes were perceived as optimal requiring no adjustments, hence this product could be scaled up by food producers but potential changes in flavor during storage should be further investigated. MLR and MLP were the most penalized juices, especially the sweetness, aroma, acidity for MLR; and sweetness, aroma, acidity consistency, flavor, and color for MLP. Hence, these attributes should be modified during reformulation. Consumers disagreed on the ideal intensity of MLP flavor, and this polarity may be attributed to the quality of this attribute rather than its quantity [53]. Furthermore, the use of just-about-right scales has been found to make respondents more aware and critical of imperfections in samples [53].

Consumers rely on sensory attributes to purchase foods and make a re-purchase on products that they like. This study showed that MLB and MPP had a higher purchasing intent than MLC, MLP, and MLR (Figure S2). However, it should be noted that all the samples scored above 30% on ‘would buy’ showing that they would compete favorably on the market. In addition to volatiles, other non-volatile compounds such as sugars and organic acids may be accumulated or depleted by lactic acid bacteria during fermentation, affecting the sensory characteristics of fermented juices.

## 5. Conclusions

Following the lactic acid bacteria fermentation, the content of sesquiterpenes, aldehydes, alcohols, and esters decreased while ketones and furans increased in mango juice. The control, mango juice fermented by *Pediococcus. pentosaceus* and *Levilactobacillus brevis* had higher amounts of ethyl acetate, ethyl butyrate, 2-hexenal, 2,6-nonadienal, 2,2-dimethylpropanal, β-selinene, γ-gurjunene, α-copaene, and δ-cadinene, while juice fermented with *Lacticaseibacillus casei*, *Lactiplantibacillus plantarum* subsp. *plantarum*, and *Lacticaseibacillus rhamnosus* had higher amounts of 2,3-butanedione and a cyclic hydrocarbon derivate. There was an association between the volatile compounds of the fermented mango juices and their sensory acceptability. Fermentation of mango juice with *Levilactobacillus brevis*, *Lacticaseibacillus casei, Lactiplantibacillus plantarum* subsp. *plantarum*, and *Pediococcus. pentosaceus* except *Lacticaseibacillus rhamnosus* did not affect the overall liking. Overall liking was related to ‘mango aroma’, ‘natural taste’, ‘sweet’, and ‘mango flavor’. Mango juices fermented by *Levilactobacillus brevis* were most accepted and are a potential product for scaling up. However, juices fermented by *Lactiplantibacillus plantarum* subsp. *plantarum* and *Lacticaseibacillus rhamnosus* were most criticized and require modifications/reformulation. A follow-up study is recommended to confirm if the changes made are effective in improving these mango products. Moreover, the flavor of mango juice fermented by *Lactiplantibacillus plantarum* subsp. *plantarum* received conflicting consumer critics, hence an appropriate consumer group should be targeted during product development and marketing. Further research may be carried out to investigate the effect of non-volatile compounds on the sensory acceptability of fermented mango juices.

## Figures and Tables

**Table 1:** Viable cell counts (log CFU/mL) of lactic acid bacteria in mango juice after 24 h fermentation.

Mango Juice Sample	Lactic Acid Bacteria Strain	T_0_	T_24_	*p*-Value
MLB	*Levilactobacillus brevis* LMG11437	7.52 ± 0.38 b	9.16 ± 0.26 a	0.039
MLC	*Lacticaseibacillus casei* LMG6904	7.22 ± 0.26 a	8.25 ± 0.87 a	0.251
MLP	*Lactiplantibacillus plantarum* subsp.* plantarum* LMG6907	7.31 ± 0.04 b	8.97 ± 0.10 a	0.002
MLR	*Lacticaseibacillus rhamnosus* LMG25859	7.04 ± 0.25 b	8.83 ± 0.48 a	0.043
MPP	*Pediococcus pentosaceus* LMG10740	7.62 ± 0.34 a	8.72 ± 0.50 a	0.125

Results are expressed as mean ± SD. Mango juice fermented with MLB—*Levilactobacillus brevis*; MLC—*Lacticaseibacillus casei*; MLP—*Lactiplantibacillus plantarum* subsp. *plantarum*; MLR—*Lacticaseibacillus rhamnosus*; MPP—*Pediococcus pentosaceus*; a, b values within rows with different lowercase letters differ significantly at *p * < 0.05. *n* = 3.

**Table 2:** Volatile compounds in the control and fermented mango juices expressed as ng/mL of 2-octanol equivalents.

Volatile Compound	RT ^a^	RI ^b^	ID ^c^	Samples ^d^						*p*-Value	Odor Quality ^e^
				Control	MLB	MLC	MPP	MLP	MLR		
Alcohols											
Ethanol	3.05	924.0	RI, MS	301 ± 11.0	295 ± 28.2	317 ± 3.83	314 ± 16.0	294 ± 9.12	281 ± 8.12	0.089	sweet
1-Hexanol	14.74	1339.8	RI, MS	24.9 ± 2.01 a	25.7 ± 1.87 a	23.3 ± 2.36 a	25.9 ± 1.31 a	27.9 ± 1.31 a	9.19 ± 2.70 b	<0.001	resin, flower, green
3-Hexen-1-ol	15.57	1365.7	RI, MS	7.58 ± 0.54 a	7.21 ± 0.45 ab	6.92 ± 0.58 ab	7.43 ± 1.20 a	7.50 ± 0.19 a	5.60 ± 0.57 b	0.027	grass
Aldehydes											
Unknown aldehyde	3.59	958.8	RI, MS	7.77 ± 0.49 b	10.3 ± 0.55 ab	7.21 ± 0.84 b	17.0 ± 2.92 a	13.0 ± 5.59 ab	9.92 ± 0.28 ab	0.006	-
2-Hexenal	10.26	1196.1	RI, MS	7.39 ± 0.67 a	3.68 ± 0.34 b	1.72 ± 0.82 c	4.32 ± 0.28 b	1.66 ± 0.32 c	0.83 ± 0.28 c	<0.001	apple, green
2,6-Nonadienal	22.08	1555.6	RI, MS	17.7 ± 4.87 c	2.90 ± 0.3 cb	1.98 ± 0.9 cb	2.89 ± 0.6 cb	0.75 ± 0.1 cb	0.60 ± 0.0 cb	<0.001	cucumber
Esters											
Ethyl acetate	2.50	897.2	RI, MS	84.7 ± 4.03 ab	80.9 ± 10.3 ab	71.0 ± 5.43 ab	87.7 ± 5.97 a	80.5 ± 6.20 ab	63.3 ± 1.69 b	0.017	pineapple
Ethyl butyrate	4.89	1022.7	RI, MS,	33.8 ± 1.60	32.8 ± 1.60	28.3 ± 4.66	31.4 ± 5.46	31.9 ± 1.22	29.0 ± 0.98	0.385	apple
Linalyl propanoate	26.19	1676.4	RI, MS	2.86 ± 0.57 b	3.88 ± 0.98 b	5.09 ± 0.08 ab	4.05 ± 0.78 b	4.74 ± 1.23 ab	7.38 ± 1.98 a	0.006	citrus-like
Ethyl dodecanoate	31.47	1832.1	RI, MS,	4.37 ± 0.56 ab	4.02 ± 0.11 ab	3.51 ± 0.19 b	4.43 ± 0.45 ab	4.85 ± 0.27 a	3.98 ± 0.36 ab	0.011	waxy
Furan											
2-Pentylfuran	10.78	1212.8	RI, MS	85.1 ± 4.65	98.9 ± 13.9	115 ± 0.13	90.5 ± 14.3	92.1 ± 16.9	92.3 ± 18.5	0.165	green bean, butter
Ketone											
2,3-Butanedione	3.53	954.9	RI, MS,	8.69 ± 1.15 b	8.36 ± 0.45 b	86.6 ± 10.9 a	5.43 ± 0.04 b	11.9 ± 0.66 b	84.2 ± 1.63 a	<0.001	butter
Monoterpenes											
α-Pinene	4.59	1012.4	RI, MS	1929 ± 103	1883 ± 83.2	2053 ± 51.1	1941 ± 183	1980 ± 111	1922 ± 123	0.600	pine, turpentine
α-Fenchene	5.35	1038.5	RI, MS	8.27 ± 0.33	8.12 ± 1.02	7.31 ± 0.31	8.08 ± 1.40	8.51 ± 1.23	7.83 ± 0.20	0.668	camphor
Camphene	5.52	1044.4	RI, MS	54.3 ± 2.82	52.8 ± 2.97	52.8 ± 5.14	54.3 ± 6.04	55.4 ± 1.22	50.7 ± 1.12	0.702	camphor
β-pinene	6.53	1079.1	RI, MS	418 ± 71.7	429 ± 31.0	333 ± 10.2	421 ± 97.5	339 ± 19.1	366 ± 42.8	0.163	pine, resin, turpentine
δ-3-Carene	8.26	1134.5	RI, MS,	7326 ± 258 ab	6822 ± 338 b	7732 ± 106 a	7576 ± 247 ab	7493 ± 211 ab	6881 ± 552 ab	0.019	lemon, resin
α-Phellandrene	8.58	1144.4	RI, MS	250 ± 19.6	240 ± 13.5	276 ± 12.2	264 ± 7.85	301 ± 67.5	228 ± 4.13	0.107	turpentine, mint, spice
β-Myrcene	8.76	1149.9	RI, MS	726 ± 35.7 ab	689 ± 32.2 b	862 ± 37.7 a	789 ± 52.6 ab	842 ± 76.8 a	703 ± 55.2 b	0.004	balsamic, must, spice
α-Terpinene	9.04	1158.5	RI, MS	138 ± 6.93 ab	131 ± 5.00 b	147 ± 1.04 a	144 ± 5.16 ab	146 ± 2.15 a	131 ± 6.99 b	0.005	pine, plastic
Limonene	9.67	1177.9	RI, MS,	358 ± 13.1 ab	340 ± 13.4 b	399 ± 14.3 a	364 ± 11.6 ab	374 ± 7.95 ab	343 ± 31.1 b	0.010	lemon, orange
β -phellandrene	9.90	1185.0	RI, MS	334 ± 19.4 ab	321 ± 11.2 ab	354 ± 1.22 a	337 ± 16.9 ab	346 ± 14.3 ab	310 ± 19.3 b	0.039	pepper, turpentine, wood
γ-Terpinene	11.13	1224.1	RI, MS,	94.2 ± 6.06	92.8 ± 4.56	102 ± 0.31	95.3 ± 5.21	99.2 ± 1.90	93.8 ± 4.46	0.135	gasoline, turpentine
β-Ocimene	11.44	1234.2	RI, MS	15.8 ± 1.00 b	16.2 ± 0.28 b	19.4 ± 0.62 b	17.2 ± 2.36 b	24.3 ± 1.68 a	18.3 ± 3.01 b	0.001	sweet, herb
P-Cymene	11.83	1246.9	RI, MS	159 ± 5.96 ab	152 ± 4.54 b	180 ± 0.97 a	166 ± 12.2 ab	167 ± 10.1 ab	151 ± 12.6 b	0.015	spice, fragrant
P-Cymenene	16.94	1407.8	RI, MS	29.4 ±0.88 a	29.0 ± 0.98 a	21.3 ± 1.89 b	29.0 ± 4.82 a	19.0 ± 2.70 b	28.2 ± 8.40 a	0.033	spice, wood, terpenic
α-Terpinolene (1)	12.09	1255.3	RI, MS	55.0 ± 2.20	52.9 ± 2.46	56.6 ± 2.09	55.7 ± 2.72	57.2 ± 1.51	52.7 ± 2.89	0.167	rosin
α-Terpinolene (2)	12.30	1262.1	RI, MS,	518 ± 31.0	498 ± 24.2	555 ± 18.0	543 ± 14.3	517 ± 14.3	503 ± 24.4	0.050	rosin
Sulphur											
Dimethyl sulfide	1.82	888.4	RI, MS	13.8 ± 1.86	13.0 ± 2.37	10.7 ± 1.33	12.2 ± 2.76	11.8 ± 0.93	11.2 ± 0.56	0.355	cabbage-like
Trihalomethane											
Chloroform	4.32	1003.1	RI, MS	170 ± 6.43 a	161 ± 22.8 a	134 ± 25.3 ab	103 ± 6.40 b	164 ± 11.4 a	138 ± 9.86 ab	0.002	sweet
Sesquiterpenes											
α-Copaene	19.01	1467.9	RI, MS,	38.9 ± 1.11	35.3 ± 2.76	35.9 ± 4.38	39.4 ± 3.06	33.6 ± 4.67	33.3 ± 4.23	0.241	wood, spice
α-Gurjunene	20.24	1503.5	RI, MS	62.8 ± 2.04	56.7 ± 4.77	66.4 ± 5.99	66.1 ± 8.80	59.9 ± 3.95	53.8 ± 6.76	0.108	wood, balsamic
Unknown Sesquiterpene	21.21	1531.0	RI, MS	13.9 ± 0.36 c	14.6 ± 1.31 bc	17.0 ± 0.16 bac	15.0 ± 1.31 bac	20.3 ± 3.59 a	19.8 ± 2.60 ba	0.006	-
α-Guaiene	22.36	1563.5	RI, MS,	7.38 ± 0.05	6.48 ± 0.43	6.43 ± 0.49	7.18 ± 0.57	5.93 ± 1.18	5.90 ± 1.28	0.185	wood, balsamic
β-Caryophyllene	22.43	1565.5	RI, MS	15.7 ± 0.46	13.9 ± 0.44	17.3 ± 1.67	16.4 ± 2.16	14.9 ± 1.07	14.2 ± 0.43	0.038	wood, spice
α-Humulene	24.87	1636.7	RI, MS	24.3 ± 1.06	21.6 ± 1.02	25.5 ± 2.40	25.5 ± 3.47	23.0 ± 1.07	22.5 ± 1.10	0.129	wood
γ-Gurjunene	25.19	1646.3	RI, MS	8.04 ± 0.23	6.98 ± 0.60	6.82 ± 0.24	7.95 ± 0.58	6.47 ± 1.12	7.04 ± 1.03	0.105	musty
α-Muurolene	25.28	1649.0	RI, MS,	25.6 ± 0.98	22.6 ± 0.90	27.7 ± 3.20	27.2 ± 4.01	24.7 ± 1.30	23.5 ± 0.75	0.094	wood
α-Amorphene	25.65	1660.1	RI, MS	7.08 ± 0.44	6.41 ± 0.49	7.42 ± 0.65	7.49 ± 0.97	0.67 ± 0.55	6.54 ± 0.67	0.268	-
β-Selinene	26.58	1688.1	RI, MS	103 ± 8.65	88.9 ± 3.33	109 ± 13.2	108 ± 9.48	95.2 ± 4.40	95.9 ± 4.95	0.060	herbal
α-Selinene	26.77	1693.8	RI, MS,	19.0 ± 1.43	16.6 ± 1.06	19.7 ± 2.03	20.4 ± 2.57	17.9 ± 1.15	18.17 ± 0.80	0.138	thyme, medicine
δ-Cadinene	27.99	1729.8	RI, MS	34.3 ± 2.79	30.4 ± 2.07	36.5 ± 3.99	36.50 ± 5.14	32.5 ± 2.35	32.3 ± 1.91	0.208	wood
Calamenene	30.42	1801.3	RI, MS	10.4 ± 0.64	9.41 ± 0.92	10.3 ± 0.62	11.0 ± 2.40	9.99 ± 1.21	9.91 ± 0.94	0.759	herb, spice
Other											
Cyclic hydrocarbon derivate	28.67	1749.8	RI, MS	0.00 ± 0.00 b	2.77 ± 0.52 a	2.08 ± 0.27 a	2.44 ± 0.27 a	2.03 ± 0.72 a	1.97 ± 0.43 a	<0.001	-

Results are expressed as mean ± SD. a, b, c, d values within rows with different lowercase letters differ significantly at *p* < 0.05. *n* = 3. ^a^ Retention time (min). ^b^ Retention indices according to the equation proposed by Vandendool & Kratz [21]. ^c^ ID, volatiles were identified according to abbreviations: RI, comparing retention indices on a ZB-Wax-column with those in the literature; MS, mass spectrum comparisons with those in the Wiley275 library. ^d^ Mango juice fermented with MLB—*Levilactobacillus brevis*; MLC—*Lacticaseibacillus casei*; MLP—*Lactiplantibacillus plantarum* subsp. *plantarum*; MLR—*Lacticaseibacillus rhamnosus*; MPP—*Pediococcus pentosaceus*. Control is mango juice with no lactic acid bacteria under the same conditions of fermentation (24 h). ^e^ Odor descriptions were cited from www.flavornet.org (accessed on 8 January 2022).

**Table 3:** Mean ± SD scores (9-point hedonic scale) for overall liking and sensory attributes of the control and fermented mango juices.

Sample	Overall Liking	Appearance	Color	Aroma	Flavor	Consistency	Acidity	Sweetness
Control	7.72 ± 1.17 ab	8.00 ± 1.20	8.06 ± 1.02	7.36 ± 1.43 ab	7.51 ± 0.76 a	7.46 ± 1.44 a	7.11 ± 1.65	7.56 ± 1.50 a
MLB	7.69 ± 0.94 a	8.10 ± 0.92	8.14 ± 0.71	7.63 ± 1.18 a	7.66 ± 0.77 a	7.45 ± 1.17 a	7.06 ± 1.41	7.63 ± 1.30 a
MLC	7.39 ± 1.11 abc	8.01 ± 1.00	7.98 ± 0.95	7.11 ± 1.51 abc	7.16 ± 0.87 ab	6.81 ± 1.80 acb	6.89 ± 1.85	7.15 ± 1.60 ab
MLP	7.03 ± 1.71 bc	7.98 ± 1.01	8.03 ± 0.84	6.79 ± 1.59 bc	6.65 ± 0.85 b	6.65 ± 1.81 bc	6.56 ± 1.81	6.96 ± 1.76 ab
MLR	6.71 ± 1.60 c	7.76 ± 1.42	7.94 ± 0.88	6.61 ± 1.66 c	6.61 ± 1.00 b	6.54 ± 1.63 c	6.28 ± 2.15	6.58 ± 1.98 b
MPP	7.51 ± 1.39 ab	7.94 ± 1.72	7.89 ± 1.44	7.43 ± 1.52 ab	7.55 ± 0.67 a	7.35 ± 1.64 ab	6.95 ± 1.79	7.61 ± 1.51 a
*p*-value	<0.001	0.589	0.517	<0.001	<0.001	<0.001	0.039	<0.001

Mango juices fermented with MLB—*Levilactobacillus brevis*; MLC—*Lacticaseibacillus casei*; MLP—*Lactiplantibacillus plantarum* subsp. *plantarum*; MLR—*Lacticaseibacillus rhamnosus*; MPP—*Pediococcus pentosaceus*; a, b, c values along a column with different lowercase letters differ significantly at *p* < 0.05 using repeated-measures ANOVA with Bonferroni post-hoc tests. Control is mango juice with no lactic acid bacteria under the same conditions of fermentation (24 h). Hedonic scale ranging from 1 = extremely dislike to 9 = extremely like. *n* = 80.

## Data Availability

The authors confirm that the data supporting the findings of this study are available within the article (and/or) its Supplementary Materials.

## Supplementary Materials

The following supporting information can be downloaded at: https://www.mdpi.com/article/10.3390/foods11030383/s1, Figure S1: Chromatogram of the control juice (pasteurized mango juice with no lactic acid bacteria under the same conditions of fermentation (24 h)); Figure S2: Purchase intent (% respondents, *n* = 80). Mango juices fermented with MLB—*Levilactobacillus brevis*; MLC—*Lacticaseibacillus casei*; MLP—*Lactiplantibacillus plantarum* subsp. *plantarum*; MLR—*Lacticaseibacillus rhamnosus*; MPP—*Pediococcus pentosaceus*. Control is mango juice with no lactic acid bacteria under the same conditions of fermentation (24 h); Table S1: Growth of different lactic acid bacteria (log CFU/mL) in mango juice after 24 h fermentation; Table S2: Socio-demographic information of the consumers (*n* = 80); Table S3: Chemical structures of the terpene family ^a^ [54,55,56,57].
