# Supplementary material for: Effect of Lactic Acid Fermentation on Volatile Compounds and Sensory Characteristics of Mango (Mangifera indica) Juices
Source: Foods. 2022 Jan 28;11(3):383. doi: 10.3390/foods11030383 (PMC8834145; doi:10.3390/foods11030383)
Supplement: Supplementary file 1 [file foods-11-00383-s001.zip › foods-1570464-supplementary.pdf]

foods

Table S1. Growth of different lactic acid bacteria (log CFU/mL) in mango juice after 24 h fermentation.

**Strain**	**0 h**	**24 h**	**P value**
Lactobacillus acidophilus	6.81 ± 0.32b	7.65 ± 0.22a	0.044
Lactiplantibacillus plantarum subsp. *plantarum*	7.48 ± 0.14b	8.94 ± 0.17a	0.003
Lacticaseibacillus rhamnosus	7.44 ± 0.08b	8.21 ± 0.24b	0.040
Lacticaseibacillus. casei	7.30 ± 0.25	8.03 ± 0.25	0.111
Levilactobacillus brevis	7.41 ± 0.16b	8.21 ± 0.23a	0.047
Leuconostoc mesenteroides	7.50 ± 0.02	7.58 ± 0.05	0.118
Pediococcus pentosaceus	7.36 ± 0.08b	8.08 ± 0.23a	0.014
Lactobacillus fermentum	6.36 ± 0.53	7.41 ± 0.29	0.124
Lactobacillus johnsonii	5.95 ± 0.13a	< 1b	<0.001
Limosilactobacillus reuteri	6.71 ± 0.62	7.56 ± 0.54	0.145

Results are expressed as mean ± SD. a, b values within rows with different lowercase letters differ significantly at $p<0.05$. $n = 3$.

Table S2. Socio-demographic information of the consumers ($n = 80$).

**Characteristic**	**Category**	***n***	**%**
Age groups (years)	18 - 25	14	17.5
	26 - 33	40	50.0
	34 - 41	22	27.5
	42 - 49	4	5.00
Gender	Female	36	45.0
	Male	44	55.0
Pay attention to diet	Yes	49	61.3
	No	31	38.8
Frequency of fruit consumption	Once a week	16	20.0
	More than once a week	41	51.3
	More than once a month but less than every week	16	20.0
	Less than once a month	7	8.80

Table S3. Chemical structures of the terpene family[a].

Monoterpenes

α-Pinene

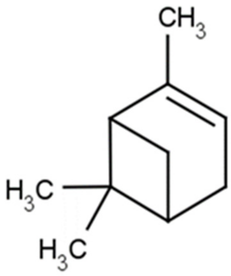

α-Fenchene

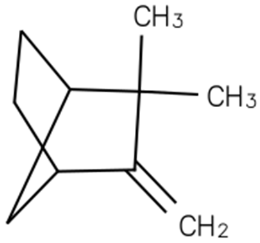

Camphene

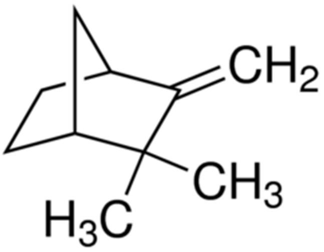

δ-3-Carene

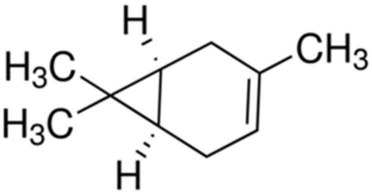

α-Phellandrene

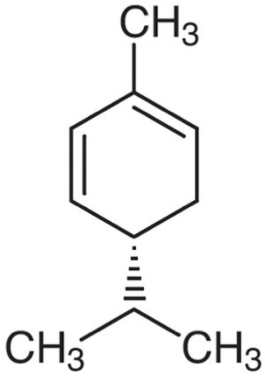

β-pinene

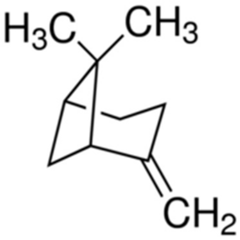

β-Myrcene

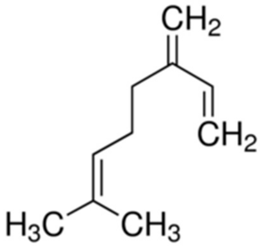

α-Terpinene

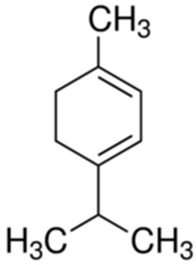

Limonene

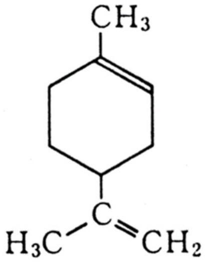

γ-Terpinene

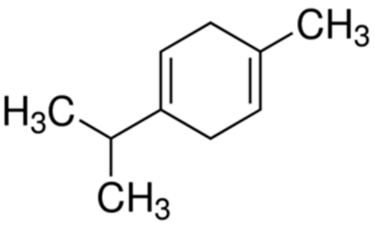

β-Ocimene

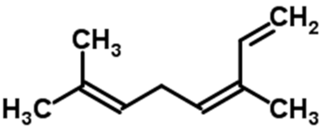

b-phellandrene

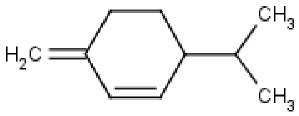

P-Cymene	P-Cymenene	α-Terpinolene
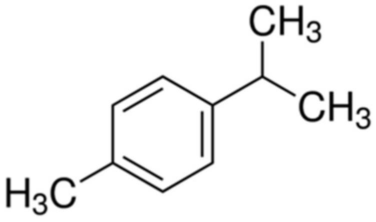	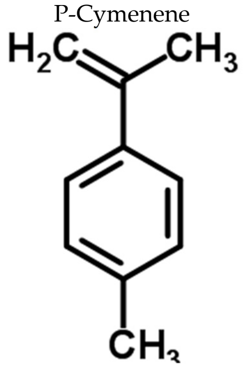	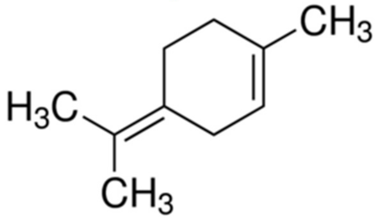
	**Sesquiterpenes**	
α-Copaene	α-Gurjunene	α-Guaiene
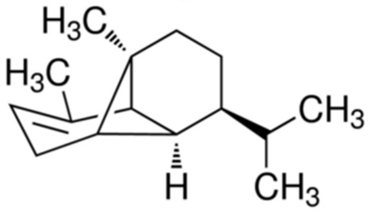	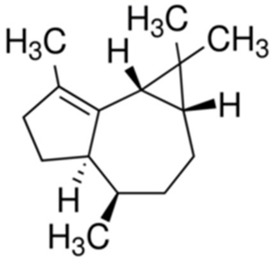	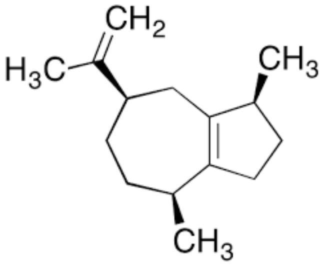
β-Caryophyllene	α-Humulene	γ-Gurjunene
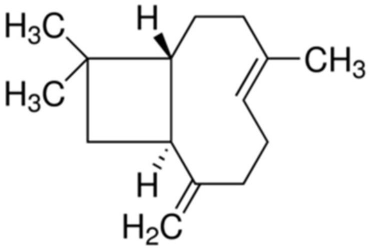	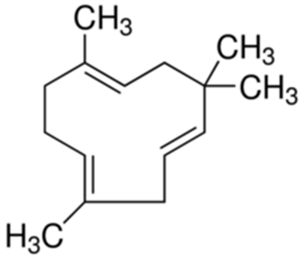	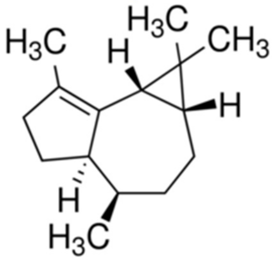
α-Muurolene	α-Amorphene	β-Selinene
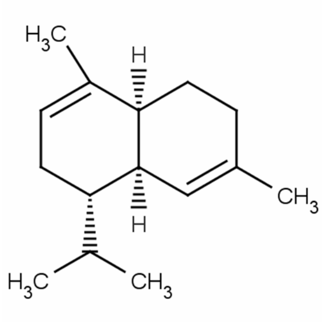	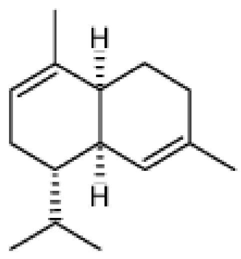	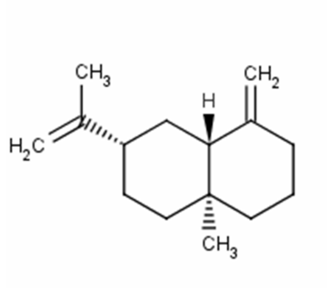

α-Selinene	δ-Cadinene	Calamenene
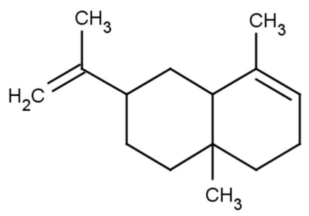	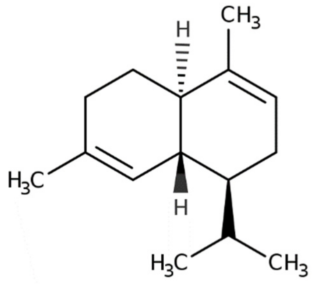	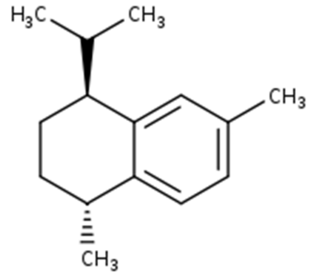

[a] References; [1–4].

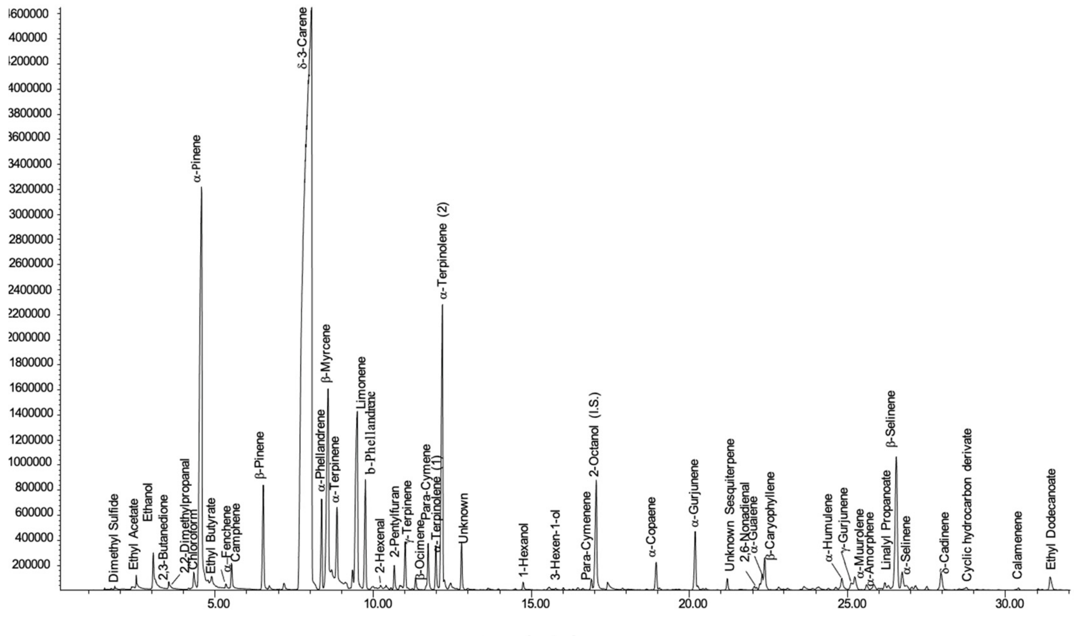

Figure S1. Chromatogram of the control juice (pasteurized mango juice with no lactic acid bacteria under the same conditions of fermentation (24 h)).

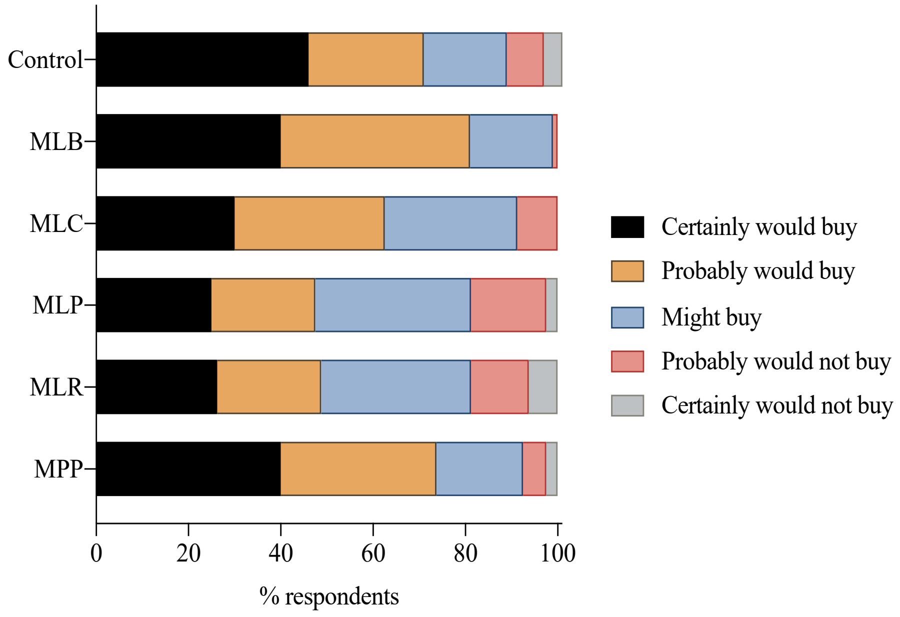

Figure S2. Purchase intent (% respondents, *n* = 80). Mango juices fermented with MLB—*Levilactobacillus brevis*; MLC—*Lacticaseibacillus casei;* MLP—*Lactiplantibacillus plantarum* subsp. *plantarum;* MLR—*Lacticaseibacillus rhamnosus;* MPP—*Pediococcus pentosaceus*. Control is mango juice with no lactic acid bacteria under the same conditions of fermentation (24 h).

References

1. Zielińska-Błajet, M.; Feder-Kubis, J. Monoterpenes and their derivatives—Recent development in biological and medical applications. *Int. J. Mol. Sci.* **2020**, *21*, 1–38. https://doi.org/10.3390/ijms21197078.
2. Huang, A.C.; Sefton, M.A.; Sumby, C.J.; Tiekink, E.R.; Taylor, D.K. Mechanistic studies on the autoxidation of α-guaiene: Structural diversity of the sesquiterpenoid downstream products. *J. Nat. Prod.* **2015**, *78*, 131–145. https://doi.org/10.1021/np500819f.
3. Ehret, C.; Ourisson, G. Le gamma-Gurjunene, Structure et Configuration: Isomerisation de l'alpha-Gurjunene. *Tetrahedron* **1969**, *25*, 1785–1799.
4. Merck, K. GaA. Structure Search. 2022. Available online: https://www.sigmaaldrich.com/BE/en/product/aldrich/ (accessed on 19 January 2022).
